# Managing cancer care during the COVID-19 pandemic: brief report from a North African single center

**DOI:** 10.11604/pamj.supp.2020.35.25020

**Published:** 2020-07-29

**Authors:** Nada Benhima, Mohamed Kaakoua, Siham Alaoui Rachidi, Mohamed El Fadli, Rhizlane Belbaraka

**Affiliations:** 1 Medical Oncology Department, Mohammed VI University Hospital, Marrakech, Morocco,; 2 Faculty of Medicine and Pharmacy, Cadi Ayyad University, Marrakech, Morocco,; 3 Medical Oncology Department, Avicenne Military Hospital, Marrakech, Morocco

**Keywords:** Cancer care, cancer policy, treatment adaptation, COVID-19 pandemic, North Africa

## Abstract

The potential threat of COVID-19 pandemic on the continuity of care for cancer patients is thought to be significant. Oncologists are weighing up the balance of risks and benefits carefully when planning daily cancer care and making treatment decisions in the face of rapid change during this public health crisis. This report describes management strategies and care models that have been adopted by a single Medical Oncology department in a North Africa, Morocco.

## Report

The COVID-19 pandemic was confirmed to have spread to Africa on February 14, 2020. On March 2nd, Morocco recorded its first case. As of July 15th, the confirmed cases reached, 16262 with a total number of 259 deaths, making it the third worst-hit country on the continent. Remarkable social and public health measures were implemented in order to control the disease spread and increase the healthcare system´s capacity. However, given the reality of scarce supplies and hospital resources in the face of the pandemic in African countries, the potential threat of COVID-19 to both our elderly and immunocompromised patients, is thought to be significant. As oncologists, we are most concerned by the “distraction effect” [1] of the pandemic on the continuous care on this large, heterogeneous group of vulnerable patients affected by cancer. In our daily practice, we have been ramping up to weigh up the balance of risks and benefits carefully when planning normal routine cancer care approach and making treatment decisions. In North Africa, so far, no clear data has been reported to reflect the impact of the pandemic on the management of cancer care. Based on our local experience and on a recent review of cancer guidelines issued by the Oncology societies, we aim to describe the unprecedented response and care models that have been adopted by a single Medical Oncology department located in an overwhelmed hospital, in the hard-hit region of the country whish, as of this day, has yet no positive case confirmed.

**Review of current evidence:** in order to review the particularities of COVID-19 in adult patients with cancer, a large systematic review of current evidence has been conducted by the oncology medical department of Saint Joseph University of Beirut, on the 5th of April. A total of 223 papers were extracted, and 88 included and analyzed by a PRISMA diagram. Out of the 88 articles, six were written in French and 19 in Chinese with English abstracts. 59% originated from China and Italy (52 of 88). Most of the papers consisted of short editorials, letters, correspondence or comments. Nine case reports, one case series and ten Cohort studies were identified (retrospective, prospective or cross-sectional analysis) which included only four exclusively cancer patients [2]. Most results concluded that patients with cancer were at a higher risk of contracting COVID-19 and had a poorer prognosis. Liang et al. were the first to publish a nationwide analysis of data regarding cancer patients who are diagnosed with COVID- 19 [3]. Their Chinese cohort was the largest and the only prospective cohort of cancer patients to date. Of 1590 cases with confirmed COVID-19, 18 patients had a history of cancer (1%; 95% CI 0.61-1.65) which seems higher than the incidence of cancer in the overall Chinese population (0.29%, 285.83 per 100000 people, according to 2015 cancer epidemiology statistics [4]. The most frequent primary tumor was lung cancer. Compared with other patient groups, people affected by cancer had a 3.5 higher risk of severe COVID-19 disease (defined as the percentage of patients admitted intensive care unit, requiring invasive ventilation, or death) (39% vs 8%, p=0.0003). In this study, receiving chemotherapy or surgery in the past month was found to further increase this risk following adjustment for other variables (OR 5.34, 95% CI 1.80-16.18; P = 0.0026). A recent report from the WHO suggested that among cancer patients, vulnerable groups at risk are patients undergoing treatment for lung cancer and blood or bone marrow cancers, such as, leukemia or multiple myeloma (at any stage of disease) and stem cell transplants [5].

**Cancer guidelines during the pandemic:** globally, healthcare systems and policy responses to COVID-19 as well as emerging evidence are evolving over the course of the pandemic. European and American oncology societies had been offering formal guidance to clinicians to adjust their routine care as the pandemic goes on. Since April 1st, the European consensus guidelines have been summarized and published in Lancet oncology in English, and in “bulletin du cancer” in French. The ASCO has released new recommendations and assembled guidance from American societies for the oncology community that have been published on the Journal of Clinical Oncology. As for ongoing research projects, the US Food and Drug Administration, the US National Cancer Institute and the European Medicines Agency (EMA) have also issued guidance on managing clinical trials during the time of the pandemic. Most of these guidelines are directed toward reducing patient´s exposure to the virus, reduce the frequency of hospital admissions, including telemedicine as an alternative means to replace office visits, making clinical decisions regarding the delay of treatment with palliative intent, favoring oral drugs, shorter administration times, or larger intervals between doses.

### How did we manage to apply these recommendations?

**Outpatient and inpatient procedures:** an outpatient department (OPD) is defined as the part of the department with allotted physical and medical facilities, with regularly scheduled hours, to provide care for patients who are not registered as inpatients. During the pandemic, the OPD has been divided into three areas, “the entrance”: Using a triage screening criteria to assess suspected cases who present with symptoms suggestive of COVID-19, with a travel history or living in epidemic-plagued zones, have had close contact with a confirmed or probable case of COVID-19 in the last 14 days; “the common area” with limited number of patients and their accompanying family members allowed to enter, and the “cancer care area” for physical examination and administration of Chemotherapy treatments. Infection control measures are strictly respected, and all patients and staff are socially distanced within the 3 areas, including the chemotherapy infusion room. Furthermore, daily phone calls are made to patients planned to be admitted the following day, to ensure they do not show any upper respiratory related symptoms or fever before their attendance at hospital appointments. As for “the inpatient department”, the priority of admission is limited to patients undergoing complex chemotherapy regimens, those with severe toxicities, and patients with potentially life-threatening conditions or injuries that require immediate treatment. Further precautions are taken to provide safe patient care.

The following clinical situations have been stratified:

*Patients on systemic therapy or planned for it:* patients undergoing treatment with intent to cure: maintain adjuvant and neoadjuvant therapy as there is no direct evidence to support withholding chemotherapy, targeted therapy, or immunotherapy among this group of patients, in whom prognosis is related to the continuity of providing care. Nonetheless, treatment regimens can be adapted in many ways. It is noteworthy that delayed initiation of adjuvant chemotherapy is associated with significantly poorer overall survival and disease-free survival, as shown in the results of 2 meta-analyses after 4-week delay for breast [6], and gastric cancer surgery [7].

*Patients undergoing palliative treatment for metastatic disease:* consider delaying for second line treatment and beyond, as long as there is no rapid progress, severe organ function impairment or a life-threating injury. The first line treatment is preferably maintained. As a matter of fact, these decisions of treatment delays are determined on a case-by-case basis. It requires careful consideration of clinical features, response to treatment, and risk of the loss of the opportunity to treat, that may require further hospital admission for palliative care, and compromise the patient outcome.

*Patients who have completed their treatment and have disease under control:* provide regular medical follow-up through telehealth services as long as they show no clinical complaints suggestive of disease relapse.

*Newly diagnosed cancer patients:* individual decisions are made, based on the potential harmful effect of delaying needed cancer-related surgery or systemic treatment. When possible, we consider initiating neoadjuvant therapy after assessing clinical stage and risk classification, in the event that surgery departments are designated to treat positive COVID-19 patients.

*Patients planned for surgery:* patients with rapid progressive disease on systematic therapy, or acute complicated conditions such as colonic obstruction or perforation are considered for urgent surgery with no delay. Radical resection operations, reconstructive surgery, and surgeries in patients who have low grade tumors are deferred. When indicated, laparoscopic procedures and minimal invasive surgery are prioritized to mitigate exposure to further increased risk. Hospital stays are shortened and surgical recovery is partially carried out at home.

*Patients on radiotherapy or planned for it:* avoiding radiotherapy for patients 65 years or older a, privileging moderate hypofractionated protocols and short course radiotherapy whenever possible.

**Specific systemic treatment adaptation:** concurrent 3-weekly cisplatin based chemoradiotherapy (100 mg/m^2^, on day 1, 22 and 43) in locally advanced squamous cell carcinoma of head and neck, seems to be the optimum drug schedule, compared with weekly cisplatin (30 - 40 mg/m^2^). Several previous studies and one meta-analysis observed no difference in the 2-, 3-year OS or 1-, 2-year LRFS, with an emphasis on 5-year OS (HR=1.79, 95%C 0.97-3.31, p=0.06) for 3-weekly regimen. Weekly regimen seemed to show more grade ≥3 mucositis (RR=1.72, p=0.01) but similar hematologic toxicities.

Randomized clinical trials comparing weekly Paclitaxel with 3 weeks Paclitaxel have produced mixed results in terms of efficacy and toxicity creating controversy about the ideal dose and schedule. During the current crisis, a 3h infusion every 3 weeks is to be preferred, as no significant survival benefit between the two common schedules was shown [8]. Although weekly Paclitaxel has significantly lower risk for neutropenia and a trend toward lower risk for sensory neuropathy than 3 weekly paclitaxel. In the treatment of adjuvant and metastatic colorectal cancer, there is no significant difference in overall survival and overall response rates between the CAPOX regimen given every 3 weeks, and FOLFOX regimen given every 2 weeks, whereas neutropenia had a higher incidence in the FOLFOX group [9]. When supported by evidence, the reduction of chemotherapy doses or treatment duration represents an active alternate treatment. In an attempt to further avoid neutropenia from occurring, consider prophylactic use of granulocyte colony stimulating factors supplementation for chemotherapy regimens with high potential for high hematological toxicity.

## Conclusion

What COVID-19 outbreak lessons teach us yet again is to support and invest in evidence-based medicine and question our abilities to rapidly respond using our research workforce. In daily oncological practice, carefully reviewing potential harm and analyzing the evidence that supports cancer treatment remains crucial.
